# The prevalence of waterpipe tobacco smoking among the general and specific populations: a systematic review

**DOI:** 10.1186/1471-2458-11-244

**Published:** 2011-04-19

**Authors:** Elie A Akl, Sameer K Gunukula, Sohaib Aleem, Rawad Obeid, Philippe Abou Jaoude, Roland Honeine, Jihad Irani

**Affiliations:** 1 Department of Medicine, State University of New York at Buffalo, NY, USA; 2 Department of Family Medicine, State University of New York at Buffalo, NY, USA; 3 Department of Clinical Epidemiology and Biostatistics, McMaster University, Hamilton Canada; 4 Department of Social and Preventive Medicine, State University of New York at Buffalo, NY, USA; 5 Department of Pediatrics, Wayne State University, Detroit, MI, USA; 6 Faculty of Health Sciences, University of Balamand, Beirut, Lebanon

## Abstract

**Background:**

The objective of this study was to systematically review the medical literature for the prevalence of waterpipe tobacco use among the general and specific populations.

**Methods:**

We electronically searched MEDLINE, EMBASE, and the ISI the Web of Science. We selected studies using a two-stage duplicate and independent screening process. We included cohort studies and cross sectional studies assessing the prevalence of use of waterpipe in either the general population or a specific population of interest. Two reviewers used a standardized and pilot tested form to collect data from each eligible study using a duplicate and independent screening process. We stratified the data analysis by country and by age group. The study was not restricted to a specific context.

**Results:**

Of a total of 38 studies, only 4 were national surveys; the rest assessed specific populations. The highest prevalence of current waterpipe smoking was among school students across countries: the United States, especially among Arab Americans (12%-15%) the Arabic Gulf region (9%-16%), Estonia (21%), and Lebanon (25%). Similarly, the prevalence of current waterpipe smoking among university students was high in the Arabic Gulf region (6%), the United Kingdom (8%), the United States (10%), Syria (15%), Lebanon (28%), and Pakistan (33%). The prevalence of current waterpipe smoking among adults was the following: Pakistan (6%), Arabic Gulf region (4%-12%), Australia (11% in Arab speaking adults), Syria (9%-12%), and Lebanon (15%). Group waterpipe smoking was high in Lebanon (5%), and Egypt (11%-15%). In Lebanon, 5%-6% pregnant women reported smoking waterpipe during pregnancy. The studies were all cross-sectional and varied by how they reported waterpipe smoking.

**Conclusion:**

While very few national surveys have been conducted, the prevalence of waterpipe smoking appears to be alarmingly high among school students and university students in Middle Eastern countries and among groups of Middle Eastern descent in Western countries.

## Background

**Figure 1:**
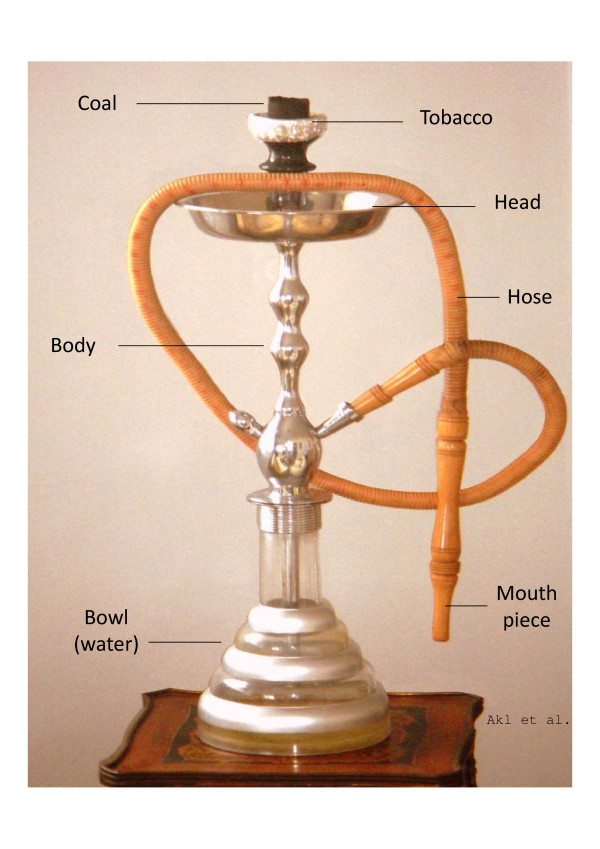
Annotated figure of a waterpipe smoking device

Tobacco smoking using waterpipe - also known as narguileh, hookah and shisha - is traditional to region of the Middle East (Figure 1)[1]. The waterpipe device heats the tobacco using charcoal, filters the resulting smoke in a bowl of water, and directs it to a rubber pipe for inhalation[2]. The type of tobacco smoked, and the shape, the size, and the appearance of the waterpipe device vary across regions[3].

A recent systematic review found that waterpipe tobacco smoking was significantly associated with lung cancer, respiratory illness, low birth weight and periodontal disease [4]. An association with bladder cancer, nasopharyngeal cancer, esophageal cancer, oral dysplasia or infertility was not ruled out [4]. Another systematic review found that waterpipe tobacco smoking negatively affects lung function and may be as harmful as cigarette smoking[5]. In spite of these deleterious health effects, waterpipe smoking is widely believed to be a less harmful form of tobacco smoking,[6] and a safer alternative to cigarette smoking[7-9].

Recent studies have reported relatively high prevalence rates of waterpipe tobacco smoking in Middle Eastern countries,[10] but also in western countries such as the United States,[2,11] the United Kingdom,[12] and Australia[13]. In 2005, the World Health Organization (WHO) issued an advisory note calling for a better understanding of national and global trends of waterpipe tobacco smoking[14]. In 2007, the American Lung Association labeled waterpipe smoking as an 'emerging deadly trend'[2]. The association called for more research on the patterns of use of waterpipe amongst various populations and to investigate its use as part of the national surveys on youth and adult tobacco use.

The primary objective of our study was to systematically review the medical literature for the prevalence of waterpipe tobacco use among the general population as well as specific populations. A secondary objective was to identify the factors associated with waterpipe tobacco use.

## Methods

No protocol for this review has been published.

### Eligibility criteria

Inclusion criteria were:

• Cohort studies and cross sectional studies

• Assessment of the prevalence of use of waterpipe for the purpose of tobacco smoking.

• The target population is either the general population or a specific population of interest such as high school students, university students, and pregnant women.

• Prevalence of waterpipe smoking reported separately from the prevalence of other forms of smoking

Exclusion criteria were:

• Convenience sampling

• Sampling methodology did not clearly lead to a representative sample of the target population (e.g., excluding cigarette smokers).

• No measure of prevalence

### Search Strategy

We electronically searched the following databases in June 2008, MEDLINE (1950 onwards), EMBASE (1980 onwards), and ISI the Web of Science using no language restrictions. The search strategy was based on related systematic review,[15] a review of eligible papers, and an Internet search for the synonyms of waterpipe (Additional file 1)[15]. We also used the 'Related Articles' feature in PubMed and reviewed the reference lists of included and relevant papers.

### Selection process

Two reviewers independently screened the title and abstract of identified citations for potential eligibility. They then used a standardized and pilot-tested form to independently screen the full texts of citations judged potentially eligible by at least one reviewer. They resolved disagreements by discussion or using an arbitrator.

### Data abstraction

Two reviewers used a standardized and pilot-tested form to independently abstract data and resolved disagreements by discussion or using an arbitrator. Abstracted data related to the following:

• Study methodology: sampling frame, sampling method, recruitment method, and administration method

• Methodological quality: sample size calculation, validity of tool, pilot testing, and response rate

• Population: country, target population, setting (location and time period), and numbers sampled, participated, and analyzed

• Results including prevalence results (ever, current, regular) of waterpipe only smoking, waterpipe smoking (regardless of other tobacco products use), cigarette smoking, and factors significantly associated with waterpipe smoking.

### Data analysis

We calculated the kappa statistic to evaluate the agreement in assessing full texts for eligibility. We stratified data analysis by country and age group. We present the results by the region of the world.

## Results

### Description of included studies

**Figure 2:**
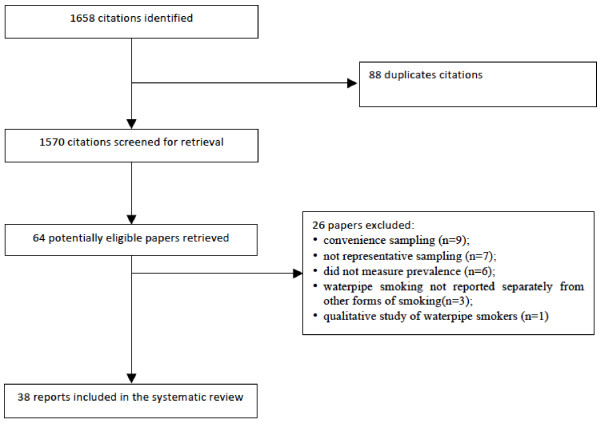
Flow of studies considered in the systematic review

Figure 2 shows the study flow and reasons for study exclusions. We considered a total of 64 studies. Of these we excluded 26 studies for the following reasons: convenience sampling (n = 9) not representative sampling (n = 7); did not measure prevalence (n = 6) waterpipe smoking not reported separately from other forms of smoking (n = 3) and qualitative study of waterpipe smokers (n = 1) (Additional file 2). We included a total of 38 studies. The countries in which the studies were conducted were: Lebanon (n = 10), Arabic Gulf countries (n = 8), USA (n = 6), Pakistan (n = 4), Egypt (n = 4), Syria (n = 3), Australia (n = 1), UK (n = 1), and Estonia (n = 1). The target populations in the identified studies were middle or high school students (n = 11), university students (n = 8), adults (n = 17), and pregnant women (n = 3). Only 26 of the 38 papers reported the year of data collection. All of the papers were published after 1998.

The studies varied by whether they reported the prevalence of waterpipe only smokers (n = 5), all waterpipe smokers (n = 37) (i.e., irrespective of whether they smoked other forms of tobacco), and by the types of measure of waterpipe smoking: ever smoker (n = 16), current smoker (n = 34), and regular smoker (n = 3). Five studies reported on group waterpipe smoking while 35 studies reported on individual smoking.

### Methodological quality of included studies

Ten studies reported sample size calculation. The instruments used to measure waterpipe use were: self developed instrument with no validation reported (n = 15); self developed instrument based on previously validated instruments, with no validation of the new instrument reported (n = 7); self developed validated instrument (n = 1); previously developed instrument with no validation reported (n = 1); previously validated instrument that measured "forms of smoked tobacco products other than cigarettes" which the authors assumed to be waterpipe smoking (n = 1) and not reported (n = 13). Fifteen studies reported pilot testing the measurement instrument. Twenty-nine studies reported response rate that varied from 18% to 62% for online administered surveys, 46% to 100% for paper-based surveys, and 70% for telephone survey.

**Figure 3:**
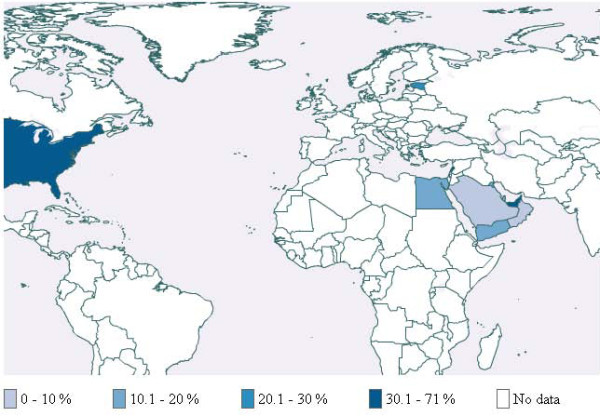
Waterpipe use among school students

**Figure 4:**
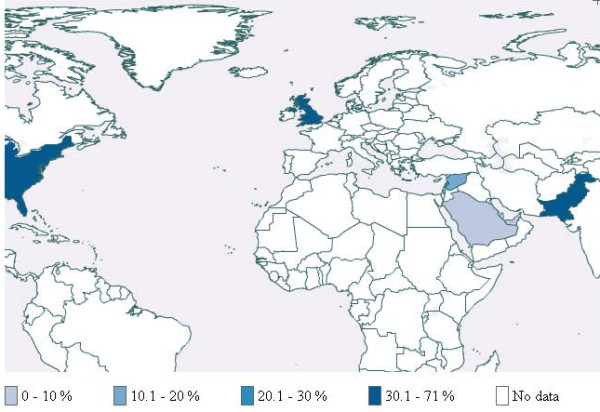
Waterpipe use among university students

**Figures 5:**
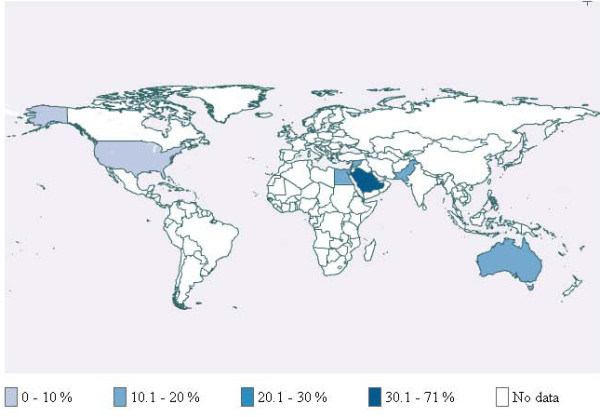
Waterpipe use among adults

Additional file 3 provides detailed description of the characteristics of included studies by world region. Table 1 provides a summary description of these studies. Figures 3, 4 and 5 respectively present the prevalence for school students, university students and adults across countries for which published studies were identified. In cases where more than one study was available, we used medians. In most of these cases, the values relate to specific populations in that country and not to the general population. The following paragraphs of the results section provide synthesized information by region.

**Table 1:** Summary of included studies

Study & setting	Population	Waterpipe use prevalence
***Middle East***		

Tamim 2003 [23]	Parents of school students (mean age 13,52% males); N = 625	Waterpipe only: 9%
Lebanon, 2000		Cigarettes & waterpipe: 18%

Bachir 2008 [39]	Pregnant women delivering in hospitals (mean age 28); N = 934	Life time: 12%
Lebanon, 1997-1998		During pregnancy: 6%

Riachy 2008 [63]	General population; N = 37579	Waterpipe only: 4%
Lebanon, 2003-2005		More than one form: 20%

Chaaya 2004 [22]	Sophomore university students (52% males); N = 416	Current: 28%
Lebanon		Ex smokers: 15%

Chaaya 2004 [41]	Pregnant women in primary care clinics (mean age 27); N = 864	Before pregnancy: 7%
Lebanon, 2003		During pregnancy: 4%

Zoughaib 2004 [17]	Teenage students (mean age 16; 57% males); N = 1461	Ever: 66%
Lebanon, 2002		Regular: 24%

Chaaya 2003 [40]	Pregnant women delivering in hospitals (mean age 28.1); N = 576	Ever: 18%
Lebanon, 2000		During pregnancy: 6%

Baddoura 2001 [33]	Lebanese adults (M/F ratio 0.95; mean age 40); N = 727	Current: 15%
Lebanon, 1997		Daily: 9%

Tamim 2007 [16,18]	School students (45% males; mean age 15); N = 2443	Current: 26%
Lebanon, 2002-2003		Ever: 65%

Tamim 2003 [23]	University students (mean age 21; 41% males); N = 1964	Waterpipe only: 21.1%
Lebanon, 2000-2001		Cigarettes & waterpipe: 11%

Al-Haddad 2003[21]	Secondary school boys (mean age 16.5); N = 600	Waterpipe: 13%
Bahrain		

Memon 2000[34]	Government employees; N = 3859	Ever: 63% (men 57%; women
Kuwait, 1996		69%)

Behbehani 2004 [36]	Physicians (65% males; mean age 45); N = 1529	Kuwait: 12% (M: 17%; F: 3%)
Kuwait, Bahrain, 2000		Bahrain: 6% (M: 9%; F: 3%)

Taha 2007 [19]	Male students (mean age 17) N = 1240; male teachers (mean age 35); N = 142	Students: current 9%, former 4%; Teachers: current 4%, former 1%
Saudi Arabia, 2001		

AL-Turki 2006 [24] Saudi Arabia, 2005	Male medical students (mean age 21.8); N = 322	Ever: 8%

Milaat 1999 [35]	Female teaching staff and employees (age range 23-62); N = 299	Current: 11%
Saudi Arabia		

Mandil 2007 [25]	Students in 13 colleges (39% males; mean age 21); N = 1057	Overall: 6% (M 11%; F 3%)
UAE, 2004-2005		

Al-Mulla 2008 [20]	School students with ages 13-15; N = 32356	Bahrain: 15%, Kuwait: 16%, Oman: 9%, Qatar: 14%, Saudi Arabia: 10%, UAE: 15%, Yemen: 15%

Arabic Gulf, 2001-2004		
Ward 2006 [37] Syria, 2004	Adults residing in Aleppo (45% men; mean age 35); N = 2038	Current: 12% Occasionally 11%

Maziak 2005 [38] Syria	Adults residing in Aleppo (46% males; mean age 34);	Overall (current): 9.1% (male: 16%, female: 4%)
	N = 1021	

Maziak 2004 [9,26-28]	Aleppo University students (47% males; mean age 22); N = 587	Current: 15% (M 26%, F 5%)
Syria, 2003		Ever: 46% (M 62.6%, F 230%)

Gadalla 2003 [29]	Students of secondary schools in villages; N = 627	Lifetime: 19% (M: 26%, F: 5%)
Egypt, 2002/03		

Habib 2000 [30]	Males in households of a village; N = 1827	Group water pipe smoking: (males only) 10.6%
Egypt, 1997		

El Sadawy 2004[31]	Males in household in Sharkia Governorate; N = 782	Group water pipe smoking (males only): 14.6%
Egypt		

Medhat 2002 [32]	Male village inhabitants 5 years and older; N = 2717	Group water pipe smoking: 13% (> 30 years: 7%)
Egypt		

***South Asia***		

Jawaid 2008 [42]	University students, Karachi (60% males; mean age 21); N = 450	Current: 33%
Pakistan, 2006-2007		Ever: 54% (M 64%; F 38%)

Nisar 2007 [44]	Adults living in semi urban community (64% males); N = 157	Overall: 13%
Pakistan, 2005		

Nisar 2005 [43]	Adult females in Karachi; N = 200	Overall: 41%
Pakistan		

Alam 1998 [45]	Adults (in urban and rural areas (47%males); N = 9441	Overall: 6% (M 7%; F6%)
Pakistan, 1990-1994		

Primack 2008 [50]	University studeents (mean age 21; 34% males); N = 647	Past 30 days: 10%
USA, 2007		Ever: 41%

Weglicki 2008 [46,47]	Arab-American and non-Arab-American youth (14-18); N = 1872	Arabs (current): 17%
USA, 2004-2005		Non Arabs (current): 11%

Rice 2007 [48]	Arab and non-Arab Americans (mean age 15; 55% M); N = 1455	Arab: last 30 days 12%; regular 8%; Other: last 30 days 4%, regular 3%
USA		

Rice 2006 [11]	Adolescents (mean age 15, 52% males); N = 1671	Experimentation: 27%
USA, 2001/02		Ever by age 14: 23%

		Ever by age 18: 40%
Ward 2006 [51]	Active duty Air Force personnel; N = 20,673	Overall: 0.3%
USA, 2000 - 2002		

***Americas***		

Primack 2009 [49]	School students (48% males; median age 14); N = 6,594	Past 30 days: 4%
USA, 2004/05		Ever: 6%

***Europe***		

Jackson 2008 [12]	University students (42% males); N = 937	Ever: 38%
United Kingdom		Regular: 8%

Parna 2008 [52]	11-15 year old school students (50% males); N = 4463	Current: 21% (M 25%; F 16%)
Estonia, 2006-2007		Daily: 0.8% (M 1% F 0.3%)

***Australia***		

Carroll 2008 [13,53]	Arabic speakers in Australia; N not reported	Current: 11%
Australia, 2004		Daily: 1%

### Middle East region

#### School students

two studies of intermediate and secondary school students in Lebanon reported 25% current and 65%-66% ever waterpipe smoking (Additional file 3; Part 1)[16-18]. Waterpipe smoking was associated with male sex, [16,17] attending public schools, and secondary classes[16,18]. The prevalence of current waterpipe smoking was about 10 times that of current cigarette smoking[16-18]. Two studies of secondary school boys in Saudi Arabia reported 9% and 10% current waterpipe smoking respectively[19,20]. A study of secondary school boys in Bahrain reported 13% waterpipe smoking and 2% waterpipe only smoking[21]. A multi-national study conducted in all schools with grades of 13-15 year old students reported current waterpipe smoking prevalence of 15% in Bahrain, 16% in Kuwait, 9% in Oman, 14% in Qatar, 15% in United Arab Emirates (UAE), and 15% in Yemen[20]. The study reported that boys were significantly more likely than girls to use waterpipe in all countries except Qatar. Waterpipe smoking was significantly more common than cigarette smoking among boys and girls in the majority of countries included in this survey.

#### University students

one study from Lebanon reported 28% current waterpipe smoking and 43% ever waterpipe smoking,[22] while another study reported 21% waterpipe only smoking[23]. Waterpipe only smoking was associated with male sex, and alcohol use (> 7 pints beer/day)[23]. The prevalence of waterpipe only smoking was about 3 times that of cigarette smoking only (Additional file 3; Part 1)[22,23]. Two studies reported 8% ever waterpipe smoking among male medical students of Saudi Arabia and 6% current waterpipe smoking among students of University of Sharjah in UAE, respectively[24,25]. Compared with cigarette smoking, the prevalence of waterpipe smoking was slightly higher in Saudi Arabia and slightly lower in the United Arab Emirates. One study from Syria reported 15% current and 46% ever waterpipe smoking[9,26-28]. Waterpipe smoking was associated with male sex, cigarette smoking, number of friends smoking waterpipe only, number of friends smoking both cigarette and waterpipe, and number of waterpipes smoked daily in the house. The prevalence of cigarette smoking was slightly higher than that of waterpipe smoking. One study of students of secondary schools in villages of Egypt reported 19% ever waterpipe smoking[29]. Three studies conducted among male adults residing in villages reported 11%-15% prevalence of waterpipe smoking in groups [30-32].

#### Adults

one study from Lebanon found 15% current waterpipe smoking and 5% group waterpipe smoking[33]. One study of adults attending primary care and specialty clinics found 4% waterpipe only smoking. One study of school students found a prevalence of 9% waterpipe only smoking by their parents at home [23]. The prevalence of cigarette only smoking was about 5 times that of waterpipe only smoking (Additional file 3; Part 1). A study of government ministry employees in Kuwait found 63% ever waterpipe smoking[34]. A study of female teaching staff and employees of King AbdulAziz University of Saudi Arabia reported 11% current waterpipe smoking with moderate correlation between waterpipe smoking and cigarette smoking[35]. A study of school teachers in Saudi Arabia reported 4% current waterpipe smoking[19]. A study of adults aged 19 and above in Bahrain reported 5% current waterpipe smoking. A study of physicians in Bahrain and Kuwait reported 6% and 12% current waterpipe smoking, respectively[36]. The prevalence of cigarette smoking was 1-3 times that of waterpipe smoking. One study from Syria found a prevalence of 12% of current waterpipe use [37] while another study found a prevalence of 9% of current waterpipe smoking[38]. The prevalence of cigarette smoking was 3-4 times that of waterpipe smoking[37,38].

#### Pregnant women

3 studies reported 7%-18% ever smoking and 5%-6% waterpipe smoking during pregnancy [39-41]. The prevalence of cigarette smoking was about 3-5 times that of waterpipe smoking during pregnancy and about 3 folds before pregnancy (Additional file 3; Part 1).

### South Asia region

We identified studies conducted only in Pakistan.

#### University students

One study reported 33% current and 53% ever waterpipe smoking (Additional file 3; Part 2). Of these, 31% students reported sharing waterpipe smoking with others[42]. Both current and ever waterpipe smoking were predominant in males (p < 0.001).

#### Adults

One study targeting female adults living on an island reported 41% waterpipe smoking[43]. Another study reported 13% waterpipe smoking[44]. Another study of a nationally representative sample reported 6% current waterpipe smoking among adults aged 15 and older[45]. In the more representative sample of the two latter studies, the prevalence of cigarette smoking was about 2 times that of waterpipe smoking (Additional file 3; Part 2)[45].

### Americas region

#### School students

3 studies targeted 14-18 years old school students with high percentages of Middle Eastern descent (Additional file 3; Part 3) [10,43-45]. These 3 studies reported 12%- 15% current, 27%-36% ever, and 7%-8% regular waterpipe smoking among students of Middle Eastern descent. They reported 5%- 8% current, 11%- 21% ever, and 3%-5% regular waterpipe smoking among students of other ethnicities. Current waterpipe smoking was associated with current cigarette smoking, Arab American ethnicity, grade in school (by grade), and family waterpipe smoking [46,47]. The prevalence of current waterpipe smoking was about 2-6 folds that of current cigarette smoking among Arabs and about half of that of cigarette smoking among non Arabs [11,46-48].

One study targeting middle and high school students of the state of Arizona found 4% current and 6% ever waterpipe smoking[49]. Current waterpipe smoking was associated with grade level, attending a charter school, Asian American and white ethnicities (compared with American Indian or Alaska Native), and male sex[49].

#### University students

1 study found 10% current, and 41% ever waterpipe smoking. The prevalence of current waterpipe smoking was about half that of current smoking (Additional file 3; Part 3)[50].

#### Adults

1 study conducted on US Air Force personnel reported a prevalence of 0.3% waterpipe smoking during their participation in a one year clinical trial of smoking prevention/cessation (Additional file 3; Part 3) [51].

### European region

#### School students

A study conducted in a nationally representative sample of 11-15 year old school students in Estonia reported 21% current waterpipe smoking (Additional file 3; Part 4) [52]. The prevalence of current waterpipe smoking was about 1.5 times that of current cigarette smoking.

#### University students

A study on university students reported 38% ever waterpipe smoking, 8% regular waterpipe smoking and 5% waterpipe only smoking (Additional file 3; Part 4)[12]. Ever waterpipe smoking was associated with male gender, and cigarette smoking.

### Australia

#### Adults

A study conducted on Arabic speaking adults reported 11% current waterpipe smoking (Additional file 3; Part 5) [13,53]. Waterpipe use was associated with age group 40-59 (relative to >60), and other forms of smoking.

## Discussion

In summary, surveys found alarming prevalence of current waterpipe smoking among school students in the United States, especially among Arab Americans (12%-15%),[11,46-48] the Arabic Gulf region (9%-16%),[19-21] Estonia (21%),[52] and Lebanon (25%)[16-18]. Similarly, the prevalence of current waterpipe smoking among university students was high in the Arabic Gulf region (6%),[25] the United Kingdom (8%),[12] the United States (10%), [50] Syria (15%),[9,26-28] Lebanon (28%),[22] and Pakistan (33%)[42]. The prevalence of current waterpipe smoking among adults was the following: Pakistan (6%),[45] Arabic Gulf region (4%-12%), [19,35,36] Australia (11% in Arab speaking adults),[13,53] Syria (9%-12%),[37,38] and Lebanon (15%)[33]. Studies reported high prevalence of group waterpipe smoking in Lebanon (5%), [33] and Egypt (11%-15%) [30,31]. In Lebanon, 5%-6% pregnant women reported smoking waterpipe during pregnancy [40,41].

Our findings are consistent with national surveys conducted by the WHO and the Global Youth Tobacco Survey (GYTS) in a number of countries (Additional file 4) [54]. Also, consistently with our findings, two recent non-systematic reviews of the topic found that waterpipe tobacco smoking is increasing in prevalence worldwide with 10-20% prevalence in Arab American young adult populations in the United States [55,56].

This study has a number of strengths. First, and to our knowledge, this is the first systematic review of the prevalence of waterpipe smoking and its associated factors across countries, age groups and genders. Second, we used the Cochrane Collaboration methodology for conducting systematic reviews, i.e. using a very sensitive and comprehensive search strategy, a duplicate and independent selection process, a duplicate and independent data abstraction process, and a rigorous appraisal of the methodological quality of included studies. Third, we abstracted the data on cigarette and other forms of tobacco smoking, wherever applicable, for comparative purposes.

The study also has a number of limitations. First, only four studies were conducted at national levels[20,36,45,52]. Second, studies varied by whether they reported the prevalence of waterpipe only smokers or all waterpipe smokers. They similarly varied by the type(s) of measure of waterpipe smoking reported (e.g. current, ever, and regular). However, the studies were consistent in the definitions of these measurement types. Third, only one study used a validated tool to measure exposure to waterpipe smoking, a practice that can vary widely in terms of frequency of use, the length of each use, and quantity of tobacco per single use [57]. Third, all of the included studies were cross-sectional in design and did not allow analyses for time trends.

The prevalence of waterpipe smoking is alarmingly high, particularly among school students [11,16-19,21,29,46-49,52] and university students[12,22-28,42,50]. This prevalence is consistently comparable to cigarette smoking among school students [16-21,29,46-48,52] and university students [12,23-28,50]. The high prevalence of waterpipe smoking among the youth could have been exacerbated by a common belief that this form of smoking is less harmful than cigarette smoking [8,58]. School students are particularly vulnerable because of the additional risk of secondhand waterpipe smoking (compared with secondhand cigarette smoking) given the social acceptability of this form of smoking [23]. Pregnant women represent a similarly vulnerable group in which the prevalence is concerning because of the evidence of increased incidence of low birth weight and pulmonary problem in newborns of mothers smoking waterpipe [39-41].

Another feature that might have contributed to the rise of waterpipe smoking is its higher social acceptability compared with cigarette smoking. In many settings, particularly in Middle Eastern countries and communities, waterpipe smoking is practiced during social activities whereby family members and friends smoke together[59]. Indeed, a number of studies showed that smoking in the house, [3,26-28,38,60,61] smoking in the family [11,25,46-48] and smoking among friends [11,25-28,48] are associated with waterpipe smoking among school students and university students.

A pattern of spread of waterpipe smoking appears to be through immigration. Indeed, while Middle Eastern countries had some of the highest prevalence of waterpipe smoking [3,15-33,35-45,60-64], in certain Western countries (e.g. USA, Australia) groups of Middle Eastern descent had the highest prevalence of waterpipe smoking [11-13,46-52]. These observations suggest that immigrant groups tend to maintain some of their culture specific health behaviors such as waterpipe smoking [65,66]. Moreover, immigrant groups might also pass on these behaviors to other groups as suggested by the significant prevalence of waterpipe smoking among non-Middle Eastern groups in these Western countries [11-13,46-52]. However, this pattern has not been found in certain European countries with very high prevalence of waterpipe use such as Estonia, Denmark, Sweden, and Germany [67].

As stated above, waterpipe tobacco smoking was consistently more common in school and university students than in adults in countries in which surveys were conducted. In the absence of longitudinal data, it is not clear whether these results reflect a time trend of age specific prevalence. If age specific prevalence has not been increasing, the above observation suggests that adolescents tend to quit waterpipe smoking as they age into adulthood. If age specific prevalence has been increasing, this suggests that school and university students have been affected the most and we might see a cohort effect of increasing prevalence among adults as these younger individuals age. Indeed, there is emerging evidence that waterpipe use predicts regular cigarette smoking, at least among Danish youth [67].

## Conclusion

Public health agencies need to recognize the burgeoning prevalence of waterpipe tobacco smoking particularly among the youth and ensure the comprehensiveness of their tobacco control strategies. These strategies could include those proven to be effective with cigarette smoking, i.e., stricter regulation of advertisement and age restriction on sales. Awareness campaigns should to be tailored to the prevailing belief systems and based on and culturally sensitive assessments of the roots of waterpipe tobacco smoking. Surveillance programs would help in monitoring the effectiveness of public health policies and strategies put in place.

There is a need for longitudinal nationally representative studies to better understand the epidemiology of this new epidemic particularly in terms of the maintenance of waterpipe smoking after teen age and the role of waterpipe as a gateway for future cigarette smoking and assist public health agencies in developing and applying control policies and strategies. Qualitative studies would be extremely helpful in understanding the misconceptions about waterpipe smoking and the relationship between waterpipe smoking and the initiation of cigarette smoking particularly among the youth. On the other hand, future studies need to be of higher methodological quality and particularly use validated tools. There is also a need to standardize the methods of reporting the prevalence (waterpipe only smokers versus all waterpipe smokers) and the type(s) of measure of waterpipe smoking (e.g. current, ever, and regular).

## Competing interests

The authors declare that they have no competing interests.

## Authors' contributions

EAA contributed to drafting the protocol, designing the search strategy, developing the forms, screening, data abstraction, data analysis, and drafting of the manuscript. SKG contributed to data abstraction, data analysis, and drafting of the manuscript. RO and SA contributed to data abstraction. PAJ and RH contributed to screening. JI contributed to drafting the protocol and designing the search strategy. All authors revised the article critically for important intellectual content and approved its final version. All authors had full access to all of the data (including statistical reports and tables) in the study and can take responsibility for the integrity of the data and the accuracy of the data analysis. EAA is the guarantor. All authors read and approved the final draft.

## Pre-publication history

The pre-publication history for this paper can be accessed here:

http://www.biomedcentral.com/1471-2458/11/244/prepub

## Supplementary Material

Additional file 1 **Electronic search strategies**. Provides the detailed search strategies used in the systematic review Click here for file

Additional file 2 **Excluded studies**. Provides a list of excluded studies and the reason for their exclusion Click here for file

Additional file 3 **Tables describing the characteristics of included studies measuring waterpipe smoking prevalence by world region**. Provides tables describing the characteristics of included studies measuring waterpipe smoking prevalence by world region Click here for file

Additional file 4 **Results of the Global Youth Tobacco Survey (GYTS) and World Health Organization (WHO) surveys**. Provides the prevalence of waterpipe smoking in the GYTS and WHO surveys Click here for file

## References

[B1] Chaouachi K The medical consequences of narghile (hookah, shisha) use in the world Revue d Epidemiologie et de Sante Publique 2007 55 165 170 17446024 10.1016/j.respe.2006.12.008

[B2] An Emerging Deadly Trend: Waterpipe Tobacco Use 2007 American Lung Association

[B3] Maziak W Ward KD Afifi Soweid RA Eissenberg T Maziak W Ward KD Afifi Soweid RA Eissenberg T Tobacco smoking using a waterpipe: a re-emerging strain in a global epidemic Tobacco Control 2004 13 327 333 10.1136/tc.2004.008169 15564614 PMC1747964

[B4] Akl EA Gaddam S Gunukula SK Honeine R Abou Jaoude P Irani J The effects of waterpipe tobacco smoking on health outcomes: a systematic review International Journal of Epidemiology 2010 in press 20207606 10.1093/ije/dyq002

[B5] Raad D Gaddam S Schunemann HJ Irani J Abou Jaoude P Honeine R Akl EA Effects of waterpipe tobacco smoking on lung function: a systematic review and meta-analysis Chest 139 4 764 74 10.1378/chest.10-0991 20671057

[B6] Maziak W Eissenberg T Ward KD Patterns of waterpipe use and dependence: implications for intervention development Pharmacology, Biochemistry & Behavior 2005 80 173 179 10.1016/j.pbb.2004.10.026 15652393

[B7] Kandela P Nargile smoking keeps Arabs in Wonderland Lancet 2000 356 1175 10.1016/S0140-6736(05)72871-3 11030308

[B8] Varsano S Ganz I Eldor N Garenkin M Varsano S Ganz I Eldor N Garenkin M [Water-pipe tobacco smoking among school children in Israel: frequencies, habits, and attitudes] Harefuah 2003 142 736 741 14631902

[B9] Maziak W Eissenberg T Rastam S Hammal F Asfar T Bachir ME Fouad MF Ward KD Beliefs and attitudes related to narghile (waterpipe) smoking among university students in Syria Annals of Epidemiology 2004 14 646 654 10.1016/j.annepidem.2003.11.003 15380795

[B10] Shihadeh A Azar S Antonios C Haddad A Shihadeh A Azar S Antonios C Haddad A Towards a topographical model of narghile water-pipe cafe smoking: a pilot study in a high socioeconomic status neighborhood of Beirut, Lebanon Pharmacology, Biochemistry & Behavior 2004 79 75 82 10.1016/j.pbb.2004.06.005 15388286

[B11] Rice VH Weglicki LS Templin T Hammad A Jamil H Kulwicki A Predictors of Arab American adolescent tobacco use Merrill-Palmer Quarterly-Journal of Developmental Psychology 2006 52 327 342 10.1353/mpq.2006.0020 PMC1533871 16909165

[B12] Jackson D Aveyard P Waterpipe smoking in students: prevalence, risk factors, symptoms of addiction, and smoke intake. Evidence from one British university BMC Public Health 2008 8 174 10.1186/1471-2458-8-174 18498653 PMC2413225

[B13] Carroll T Poder N Perusco A Is concern about waterpipe tobacco smoking warranted ? Australian and New Zealand Journal of Public Health 2008 32 181 U111 10.1111/j.1753-6405.2008.00198.x 18412692

[B14] WHO study group on Tobacco Product Regualation (TobReg). Advisory Note. Waterpipe tobacco smoking: health effects research needs and recommended actions by regulators 2005 10.1186/1477-5751-5-17 PMC1664583 17112380

[B15] Maziak W Ward KD Eissenberg T Interventions for waterpipe smoking cessation Cochrane Database of Systematic Reviews 2007 CD005549 10.1002/14651858.CD005549.pub2 17943865

[B16] El-Roueiheb Z Tamim H Kanj M Jabbour S Alayan I Musharrafieh U Cigarette and waterpipe smoking among Lebanese adolescents, a cross-sectional study, 2003-2004 Nicotine & Tobacco Research 2008 10 309 314 10.1080/14622200701825775 18236295

[B17] Zoughaib SS Adib SM Jabbour J Zoughaib SS Adib SM Jabbour J Prevalence and determinants of water pipe or narghile use among students in Beirut's southern suburbs Journal Medical Libanais - Lebanese Medical Journal 2004 52 142 148 16432971

[B18] Tamim H Al-Sahab B Akkary G Ghanem M Tamim N El Roueiheb Z Kanj M Afifi R Tamim H Al-Sahab B Cigarette and nargileh smoking practices among school students in Beirut, Lebanon American Journal of Health Behavior 2007 31 56 63 17181462 10.5555/ajhb.2007.31.1.56

[B19] Taha AZA Prevalence of Risk-taking Behaviors Bahrain Medical Bulletin 2007 29 1 10

[B20] Al-Mulla AM Helmy SA Al-Lawati J Al Nasser S Rahman SAA Almutawa A Saab BA Al-Bedah AM Al-Rabeah AM Bahaj AA Prevalence of tobacco use among students aged 13-15 years in Health Ministers' Council/Gulf Cooperation Council Member States, 2001-2004 Journal of School Health 2008 78 337 343 10.1111/j.1746-1561.2008.00311.x 18489467

[B21] Al-Haddad N Hamadeh RR Al-Haddad N Hamadeh RR Smoking among secondary-school boys in Bahrain: prevalence and risk factors Eastern Mediterranean Health Journal 2003 9 78 86 15562736

[B22] Chaaya M El-Roueiheb Z Chemaitelly H Azar G Nasr J Al-Sahab B Chaaya M El-Roueiheb Z Chemaitelly H Azar G Argileh smoking among university students: a new tobacco epidemic Nicotine & Tobacco Research 2004 6 457 463 10.1080/14622200410001696628 15203779

[B23] Tamim H Terro A Kassem H Ghazi A Khamis TA Hay MM Musharrafieh U Tamim H Terro A Kassem H Tobacco use by university students, Lebanon, 2001 Addiction 2003 98 933 939 10.1046/j.1360-0443.2003.00413.x 12814499

[B24] Al-Turki YA Al-Turki YA Smoking habits among medical students in Central Saudi Arabia Saudi Medical Journal 2006 27 700 703 16680263

[B25] Mandil A Hussein A Omer H Turki G Gaber I Mandil A Hussein A Omer H Turki G Gaber I Characteristics and risk factors of tobacco consumption among University of Sharjah students, 2005 Eastern Mediterranean Health Journal 2007 13 1449 1458 18341194 10.26719/2007.13.6.1449

[B26] Maziak W Eissenberg T Rastam S Hammal F Asfar T Bachir ME Fouad MF Ward KD Maziak W Eissenberg T Beliefs and attitudes related to narghile (waterpipe) smoking among university students in Syria Annals of Epidemiology 2004 14 646 654 10.1016/j.annepidem.2003.11.003 15380795

[B27] Maziak W Fouad FM Asfar T Hammal F Bachir EM Rastam S Eissenberg T Ward KD Maziak W Fouad FM Prevalence and characteristics of narghile smoking among university students in Syria International Journal of Tuberculosis & Lung Disease 2004 8 882 889 15260281

[B28] Maziak W Hammal F Rastam S Asfar T Eissenberg T Bachir ME Fouad MF Ward KD Maziak W Hammal F Characteristics of cigarette smoking and quitting among university students in Syria Preventive Medicine 2004 39 330 336 10.1016/j.ypmed.2004.01.024 15226042

[B29] Gadalla S Aboul-Fotouh A El-Setouhy M Mikhail N Abdel-Aziz F Mohamed MK Kamal Ael A Israel E Gadalla S Aboul-Fotouh A Prevalence of smoking among rural secondary school students in Qualyobia governorate Journal of the Egyptian Society of Parasitology 2003 33 1031 1050 15119469

[B30] Habib M Mohamed MK Abdel-Aziz F Magder LS Abdel-Hamid M Gamil F Madkour S Mikhail NN Anwar W Strickland GT Hepatitis C virus infection in a community in the Nile Delta: risk factors for seropositivity Hepatology 2001 33 248 253 10.1053/jhep.2001.20797 11124843

[B31] El-Sadawy M Ragab H el-Toukhy H el-Mor Ael L Mangoud AM Eissa MH Afefy AF el-Shorbagy E Ibrahem IA Mahrous S Hepatitis C virus infection at Sharkia Governorate, Egypt: seroprevalence and associated risk factors Journal of the Egyptian Society of Parasitology 2004 34 367 384 15124747

[B32] Medhat A Shehata M Magder LS Mikhail N Abdel-Baki L Nafeh M Abdel-Hamid M Strickland GT Fix AD Hepatitis c in a community in Upper Egypt: risk factors for infection Am J Trop Med Hyg 2002 66 633 638 12201604 10.4269/ajtmh.2002.66.633

[B33] Baddoura R Wehbeh-Chidiac C Prevalence of tobacco use among the adult Lebanese population Eastern Mediterranean Health Journal 2001 7 819 828 15332785

[B34] Memon A Moody PM Sugathan TN el-Gerges N al-Bustan M al-Shatti A al-Jazzaf H Epidemiology of smoking among Kuwaiti adults: prevalence, characteristics, and attitudes Bull World Health Organ 2000 78 1306 1315 11143190 PMC2560632

[B35] Milaat WA Al-Bar HS Ghabrah TM Abalkhail BA Suliman NK Preventive practices and non healthy behaviors among female university employees in Saudi Arabia Bahrain Medical Bulletin 1999 21 3 75 79

[B36] Behbehani NN Hamadeh RR Macklai NS Behbehani NN Hamadeh RR Macklai NS Knowledge of and attitudes towards tobacco control among smoking and non-smoking physicians in 2 Gulf Arab states Saudi Medical Journal 2004 25 585 591 15138525

[B37] Ward KD Eissenberg T Rastam S Asfar T Mzayek F Fouad MF Hammal F Mock J Maziak W Ward KD The tobacco epidemic in Syria Tobacco Control 2006 15 Suppl 1 i24 29 10.1136/tc.2005.014860 16723671 PMC2563543

[B38] Maziak W Ward KD Mzayek F Rastam S Bachir ME Fouad MF Hammal F Asfar T Mock J Nuwayhid I Mapping the health and environmental situation in informal zones in Aleppo, Syria: report from the Aleppo household survey International Archives of Occupational and Environmental Health 2005 78 547 558 10.1007/s00420-005-0625-7 15999277

[B39] Bachir R Chaaya M Maternal smoking: Determinants and associated morbidity in two areas in Lebanon Maternal and Child Health Journal 2008 12 298 307 10.1007/s10995-007-0242-z 17587161

[B40] Chaaya M Awwad J Campbell OM Sibai A Kaddour A Chaaya M Awwad J Campbell OMR Sibai A Kaddour A Demographic and psychosocial profile of smoking among pregnant women in Lebanon: public health implications Maternal & Child Health Journal 2003 7 179 186 10.1023/A:1025136421230 14509413 PMC1457110

[B41] Chaaya M Jabbour S El-Roueiheb Z Chemaitelly H Chaaya M Jabbour S El-Roueiheb Z Chemaitelly H Knowledge, attitudes, and practices of argileh (water pipe or hubble-bubble) and cigarette smoking among pregnant women in Lebanon Addictive Behaviors 2004 29 1821 1831 10.1016/j.addbeh.2004.04.008 15530724

[B42] Jawaid A Zafar AM Rehman TU Nazir MR Ghafoor ZA Afzal O Khan JA Knowledge, attitudes and practice of university students regarding waterpipe smoking in Pakistan International Journal of Tuberculosis & Lung Disease 2008 12 1077 1084 18713508

[B43] Nisar N Billoo N Gadit AA Nisar N Billoo N Gadit AA Pattern of tobacco consumption among adult women of low socioeconomic community Karachi, Pakistan JPMA - Journal of the Pakistan Medical Association 2005 55 111 114 15852747

[B44] Nisar N Qadri MH Fatima K Perveen S Nisar N Qadri MH Fatima K Perveen S A community based study about knowledge and practices regarding tobacco consumption and passive smoking in Gadap Town, Karachi JPMA - Journal of the Pakistan Medical Association 2007 57 186 188 17489526

[B45] Alam SE Alam SE Prevalence and pattern of smoking in Pakistan JPMA - Journal of the Pakistan Medical Association 1998 48 64 66 9783029

[B46] Weglicki LS Templin T Hammad A Jamil H Abou-Mediene S Farroukh M Rice VH Tobacco use patterns among high school students: Do Arab American youth differ? Ethnicity & disease 2007 17 S3 22-S23-24 17985444

[B47] Weglicki LS Templin TN Rice VH Jamil H Hammad A Comparison of cigarette and water-pipe smoking by Arab and non-Arab-American youth American Journal of Preventive Medicine 2008 35 334 339 10.1016/j.amepre.2008.06.037 18675529 PMC2575814

[B48] Rice VH Templin T Hammad A Weglicki L Jamil H Abou-Mediene S Collaborative research of tobacco use and its predictors in Arab and non-Arab American 9th graders Ethnicity & Disease 2007 17 S19 S21 17985443

[B49] Primack BA Walsh M Bryce C Eissenberg T Water-Pipe Tobacco Smoking Among Middle and High School Students in Arizona Pediatrics 2009 123 e282 288 10.1542/peds.2008-1663 19171581 PMC3013632

[B50] Primack BA Sidani J Agarwal AA Shadel WG Donny EC Eissenberg TE Prevalence of and associations with waterpipe tobacco smoking among US university students Annals of Behavioral Medicine 2008 36 81 86 10.1007/s12160-008-9047-6 18719977 PMC3004534

[B51] Ward KD Vander Weg MW Relyea G Debon M Klesges RC Ward KD Vander Weg MW Relyea G Debon M Klesges RC Waterpipe smoking among American military recruits Preventive Medicine 2006 43 92 97 10.1016/j.ypmed.2006.03.010 16675003

[B52] Parna K Usin J Ringmets I Cigarette and waterpipe smoking among adolescents in Estonia: HBSC survey results, 1994-2006 BMC Public Health 2008 8 392 10.1186/1471-2458-8-392 19032756 PMC2613150

[B53] Perusco A Rikard-Bell G Mohsin M Millen E Sabry M Poder N Williams M Farag L Hua M Guirguis S Tobacco control priorities for Arabic speakers: key findings from a baseline telephone survey of Arabic speakers residing in Sydney's south-west Health Promot J Austr 2007 18 121 126 17663647 10.1071/he07121

[B54] Global Youth Tobacco Survey http://www.cdc.gov/tobacco/global/GYTS/intro.htm

[B55] Cobb C Ward KD Maziak W Shihadeh AL Eissenberg T Waterpipe tobacco smoking: an emerging health crisis in the United States. [Review] [94 refs] American Journal of Health Behavior 2010 34 275 285 20001185 10.5993/ajhb.34.3.3 PMC3215592

[B56] Maziak W The waterpipe: time for action Addiction 2008 103 1763 1767 10.1111/j.1360-0443.2008.02327.x 18778388 PMC2588474

[B57] Akl E Aleem S Gunukula S Honeine R Abou Jaoude P Irani J Survey instruments used in clinical and epidemiological research on waterpipe tobacco smoking: a systematic review BMC Public Health 2010 10 415 10.1186/1471-2458-10-415 20626899 PMC2912817

[B58] Therese Carroll NP Perusco Andrew Is concern about waterpipe tobacco smoking warranted? Australian and New Zealand Journal of Public Health 2008 32 181 182 10.1111/j.1753-6405.2008.00198.x 18412692

[B59] Chaouachi K Narghile (hookah): a Socio-Anthropological Analysis. Culture, Conviviality, History and Tobaccology of a Popular Tobacco Use Mode 2000 Université Paris X

[B60] Maziak W Smoking in Syria: profile of a developing Arab country International Journal of Tuberculosis & Lung Disease 2002 6 183 191 11934135

[B61] Maziak W Ward KD Rastam S Mzayek F Eissenberg T Extent of exposure to environmental tobacco smoke (ETS) and its dose-response relation to respiratory health among adults Respir Res 2005 6 13 10.1186/1465-9921-6-13 15701169 PMC549073

[B62] Memon A Moody PM Sugathan TN el-Gerges N al-Bustan M al-Shatti A al-Jazzaf H Epidemiology of smoking among Kuwaiti adults: prevalence, characteristics, and attitudes Bulletin of the World Health Organization 2000 78 1306 1315 11143190 PMC2560632

[B63] Riachy M Rehayem C Khoury C Safi J Khayat G Aoun-Bacha Z Saade-Riachy C Kouche N Geahchan N Are narghile smokers different from cigarette smokers? Revue Des Maladies Respiratoires 2008 25 313 318 10.1016/S0761-8425(08)71550-X 18449097

[B64] Tamim H Akkary G El-Zein A El-Roueiheb Z El-Chemaly S Tamim H Akkary G El-Zein A El-Roueiheb Z El-Chemaly S Exposure of pre-school children to passive cigarette and narghile smoke in Beirut European Journal of Public Health 2006 16 509 512 10.1093/eurpub/ckl043 16675481

[B65] Hammal F Mock J Ward K Eissenberg T Maziak W A pleasure among friends: how narghile (waterpipe) smoking differs from cigarette smoking in Syria Tobacco Control 2008 17 e3 10.1136/tc.2007.020529 18375726

[B66] Ward K Eissenberg T Rastam S Asfar T Mzayek F Fouad M The tobacco epidemic in Syria Tobacco Control 2006 15 i24 i29 10.1136/tc.2005.014860 16723671 PMC2563543

[B67] Jensen PD Cortes R Engholm G Kremers S Gislum M Waterpipe use predicts progression to regular cigarette smoking among Danish youth Subst Use Misuse 2010 45 1245 1261 10.3109/10826081003682909 20441461

