# Supplementary material for: The prevalence of waterpipe tobacco smoking among the general and specific populations: a systematic review
Source: BMC Public Health. 2011 Apr 19;11:244. doi: 10.1186/1471-2458-11-244 (PMC3100253; doi:10.1186/1471-2458-11-244)
Supplement: Additional file 1 — Electronic search strategies. Provides the detailed search strategies used in the systematic review [file 1471-2458-11-244-S1.DOC]

**Additional file 1:** Electronic search strategies

**MEDLINE** (1950 onward)

Waterpipe*.mp.

“water pipe*”.mp.

shisha*.mp.

sheesha*.mp.

hooka*.mp.

huqqa*.mp.

guza*.mp.

goza*.mp.

narghil*.mp.

nargil*.mp.

arghil*.mp

argil*.mp

(hubbl* adj3 bubbl*).mp.

or/1-13

[**EMBASE**](http://ovidsp.tx.ovid.com.login.ezproxy.library.ualberta.ca/spa/ovidweb.cgi?New+Database=Single|13&S=FOJMFPFGAPDDIMNPMCHLOAOKGFPPAA00) (1988 onward)

Waterpipe*.mp.

“water pipe*”.mp.

shisha*.mp.

sheesha*.mp.

hooka*.mp.

huqqa*.mp.

guza*.mp.

goza*.mp.

narghil*.mp.

nargil*.mp.

arghil*.mp

argil*.mp

(hubbl* adj3 bubbl*).mp.

or/1-13

**ISI the Web of Science**

(waterpipe* OR "water pipe*" OR shisha* OR sheesha* OR hooka* OR huqqa* OR guza* OR goza* OR narghil* OR nargil* OR argil* OR arghil* OR (hubbl* SAME bubbl*)) AND (smoking OR smoke OR health OR disease OR cancer* OR malignan* OR lung* OR pulmonary OR heart OR cardiac OR vascular OR stroke) (in Title or Topic)
