# Supplementary material for: The prevalence of waterpipe tobacco smoking among the general and specific populations: a systematic review
Source: BMC Public Health. 2011 Apr 19;11:244. doi: 10.1186/1471-2458-11-244 (PMC3100253; doi:10.1186/1471-2458-11-244)
Supplement: Additional file 2 — Excluded studies. Provides a list of excluded studies and the reason for their exclusion [file 1471-2458-11-244-S2.DOC]

**Additional file 2:** Excluded studies

| **Study** | **Reason for exclusion** |
| --- | --- |
| 1. Mirahmadizadeh 2007 | No representative sample: one exclusion criteria in the study was: current cigarette smokers; complicated pregnancies |
| 1. Kelishadi 2006 | Does not measure prevalence |
| 1. Merdad 2007 | Waterpipe smoking not reported separately from other forms of smoking |
| 1. Nuwayhid 1998 | Does not measure prevalence. Authors sampled water pipe and cigarette smokers to study outcomes |
| 1. Maziak 2004 | Does not measure prevalence. Authors sampled water pipe and cigarette smokers to study dependence |
| 1. Labib 2007 | Does not measure prevalence. Authors selected water pipe smokers in cafes |
| 1. Fakhfakh 2002 | Waterpipe smoking not reported separately from other forms of smoking |
| 1. Smith-Simone 2008 | Does not measure prevalence. Authors selected water pipe smokers in cafes |
| 1. Kulwicki 2007 | Waterpipe smoking not reported separately from other forms of smoking |
| 1. Richter 2006 | Qualitative study of smokers |
| 1. Tamim 2008 | No representative sample. One exclusion criteria in the study was: singleton deliveries |
| 1. Hazelton 2001 | No representative sample. One exclusion criteria in the study was: availability of complete history |
| 1. Tran 2005 | Does not measure prevalence. Randomized control trial |
| 1. Knishkowy 2007 | Authors used a convenience sampling method |
| 1. Varsano 2003 | Authors used a convenience sampling method |
| 1. Smith 2007 | Authors used a convenience sampling method |
| 1. Baker 2008 | Authors used a convenience sampling method |
| 1. Eissenberg 2008 | Authors used a convenience sampling method |
| 1. Grekin 2008 | Authors used a convenience sampling method |
| 1. Almas 2003 | Authors used a convenience sampling method |
| 1. Fadhil 2007 | Authors used a convenience sampling method |
| 1. Tamim 2006 | Authors used a convenience sampling method |
| 1. Natto 2005 | No representative sample. One exclusion criteria in the study was: at least 20 teeth; case control studies |
| 1. Natto 2004 | No representative sample. One exclusion criteria in the study was: at least 20 teeth; case control studies |
| 1. Balijoon 2004 | No representative sample. One exclusion criteria in the study was: at least 20 teeth; case control studies |
| 1. Balijoon 2005 | No representative sample. One exclusion criteria in the study was: at least 20 teeth; case control studies |
