# Supplementary material for: The prevalence of waterpipe tobacco smoking among the general and specific populations: a systematic review
Source: BMC Public Health. 2011 Apr 19;11:244. doi: 10.1186/1471-2458-11-244 (PMC3100253; doi:10.1186/1471-2458-11-244)
Supplement: Additional file 3 — Tables describing the characteristics of included studies measuring waterpipe smoking prevalence by world region. Provides tables describing the characteristics of included studies measuring waterpipe smoking prevalence by world region [file 1471-2458-11-244-S3.DOCX]

**Additional file 3:** Tables describing the characteristics of included studies measuring waterpipe smoking prevalence by world region

- **Table 1:** Characteristics of included studies measuring waterpipe smoking prevalence in the Middle East
- **Table 2:** Characteristics of included studies measuring waterpipe smoking prevalence in South Asia
- **Table 3:** Characteristics of included studies measuring waterpipe smoking prevalence in the Americas
- **Table 4:** Characteristics of included studies measuring waterpipe smoking prevalence in Europe
- **Tables 5:** Characteristics of included studies measuring waterpipe smoking prevalence in Australia

**Table 1:** Characteristics of included studies measuring waterpipe smoking prevalence in the Middle East

| **Study ID** | **Methodology** | **Population/Setting** | **Prevalence Results** |
| --- | --- | --- | --- |
| Tamim 2003 [34] | - Sampling method: sampling of representative private schools - Validity of tool: not reported - Response rate: 100% | - Country: Lebanon - Population: 2nd and 3rd intermediate class students (mean age 13,51.6% males) - Setting: classroom, Apr 2000 - N sampled: 625 - N participated: 625 - N analyzed: 625 | Smoking habits of parents:   - Waterpipe only: 8.5% - Cigarettes only: 43.2% - Cigarettes or waterpipe: 70.1% - Cigarettes and water pipe: 18.4% |
| Bachir 2008 [40] | - Sampling method: random sampling - Validity of tool: not reported - Response rate: 100% (phase 1), 73.6% (phase 2) | - Country: Lebanon - Population: pregnant women delivering in hospitals situated in Beirut and Bekaa valley (mean age 28. 2) - Setting: Beirut (Jun - Aug 1998) and Bekaa valley (Oct - Dec 1997) - N sampled: 538 - N participated:– 538 (phase 1) and 396 (phase 2) - N analyzed: 538 and 396 | - Waterpipe:   - life time: 11.9%   - during pregnancy: 6.1% - Cigarette:   - life time: 29.9%   - during pregnancy: 21.8% - Any form:   - during pregnancy: 25.7% |
| Riachy 2008 [63] (in French, summary in English) | - Sampling method: random sampling - Validity of tool: not reported - Response rate: N/A - retrospective study of medical records | - Country: Lebanon - Population: Lebanese population - Setting: specialized centers, 2003-2005 - N sampled: N/A - N participated: N/A - N analyzed: 37579 | - Waterpipe only: 4.06% - Cigarette only: 12.46% - Pipe only: 0.37% - More than one form: 19.8% |
| Chaaya 2004 [22] | - Sampling method: stratified cluster sampling; stratification by faculties or professional school; cluster was sophomore classes - Validity of tool: self developed tool, validation not reported - Response rate: 100% | - Country: Lebanon - Population: sophomore students at AUB (52% males) - Setting: location and timing not reported - N sampled: 416 - N participated: 416 - N analyzed: 416 | - Waterpipe:   - current:28.3%   - ex smokers: 14.5% - Frequency of current waterpipe use:   - daily: 5.2%   - weekly: 33%   - occasional: 61% |
| Chaaya 2004 [42] | - Sampling method: stratified random sampling; stratification by geographic area (district) and expected number of pregnant women attending the primary care center - Validity of tool: self developed validated tool - Response rate: 95% | - Country: Lebanon - Population: pregnant women presenting for prenatal care in PCC (mean age 27) - Setting: prenatal clinics, Feb-Mar 2003 - N sampled: 909 - N participated: 864 - N analyzed: 864 | - Waterpipe:   - before pregnancy: 7.4%   - during pregnancy: 4.3% - Cigarette:   - before pregnancy: 19.6   - during pregnancy: 17% - Both cigarette and waterpipe:   - before pregnancy: 2.3%   - during pregnancy: 1.4% |
| Zoughaib 2004 [17] | - Sampling method: multistage sampling involving random and cluster methods - Validity of tool: self developed tool, validation not reported - Response rate: one school refused, otherwise 100% participation rate | - Country: Lebanon - Population: teenage and intermediate school students of private and public schools in Dahia (mean age 15.7; 56.7% males) - Setting: schools classes, 2002 - N sampled: not reported - N participated: 1461 - N analyzed: 1461 | - Waterpipe:   - ever: 66%   - initiators: 17.7%   - occasional: 14%   - regular: 23.9% - Cigarette:   - ever: 10.5%   - regular: 0.5% - Waterpipe and cigarette:   - regular: 4% |
| Chaaya 2003 [41] | - Sampling method: stratified random sampling; stratification by type of hospital, number of deliveries/month, and geographic area - Validity of tool: self developed tool, validation not reported - Response rate: 98.6% | - Country: Lebanon - Population: pregnant women delivering in hospitals in Beirut and its Northern and Southern suburbs (mean age 28.1) - Setting: hospital, May 2000 - N sampled: 584 - N participated:576 - N analyzed:576 | - Waterpipe:   - ever: 18%   - during pregnancy: 6% - Cigarette:   - pre pregnancy: 28%   - during pregnancy: 19.3% |
| Baddoura 2001 [33] | - Sampling method: multistage cluster sampling; stratification by districts - Validity of tool: previously available tool (Emile Roux questionnaire), validation not reported - Response rate: 88% | - Country: Lebanon - Population: Lebanese adults aged 19 and above (male/female ration 0.95; mean age 40.1) - Setting: 1997, location not reported - N sampled:825 - N participated:727 - N analyzed:727 | - Waterpipe:   - current: 14.6%   - daily: 9.2%   - group smoking(>2 pipes/day): 4.6% - Cigarette(current): 50.9% - Cigars(current): 1.5% - Pipe tobacco (current): 1.3% - Cigarillos (current): (0.5%) |
| Tamim 2007 [16] [18] | - Sampling method: multi stage sampling - Validity of tool: not reported - Response rate: more than 95% | - Country: Lebanon - Population: all students of intermediary and secondary classes of selected school (45% males; mean age 15) - Setting: classrooms, spring semester of the academic year 2002/03 - N sampled: not reported - N participated: 2443 - N analyzed: 2443 | - Waterpipe:   - current: 25.6%   - ever: 64.9% - Cigarette:   - current: 2.5%   - ever: 37.7% - Both cigarette and waterpipe (current): 6.3% |
| Tamim 2003 [23] | - Sampling method: multistage sampling - Validity of tool: not reported - Response rate: not reported | - Country: Lebanon - Population: University students (mean age 21; 41% males) - Setting: university campus, spring semester, academic year 2000–01 - N sampled: not reported - N participated: 1964 - N analyzed: 1964 | - Waterpipe only: 21.1% - Cigarette only: 7.6% - Both cigarettes and waterpipe: 11.3% - Any form : 40% |
| Al-Haddad 2003 [21] | - Sampling method: cluster random sampling, cluster was class - Validity of tool: self developed tool, validation not reported (based on WHO guidelines for tobacco smoking surveys for young people & questions taken from a Chinese study) - Response rate: 99.7% | - Country: Bahrain - Population: secondary school boys in Bahrain (mean age 16.5) - Setting: classrooms, timing not reported - N sampled: 602 - N participated:602 - N analyzed:600 | - Waterpipe: 13% - Waterpipe only: 1.5% - Waterpipe and cigarette: 10% - Waterpipe and cigar: 1.5% - Cigarette: 21% - Cigarette only: 11% - Cigar: 1.7% - Cigar only: 0.2% |
| Memon 2000[35] | - Sampling method: three-stage stratified cluster sampling; stratification by ministry (1st stage), departments (2nd stage), and sex. Employees grouped into clusters. - Validity of tool: not reported (modified version of standard WHO questionnaire) - Response rate: 96.5% | - Country: Kuwait - Population: Kuwaitis employed in all government ministries except foreign affairs and defense - Setting: ministries, Apr to Dec 1996 - N sampled:4000 - N participated: 3859 - N analyzed: 3859 | - Waterpipe (ever):   - over all 63.4%   - men 57%   - women 69% - Cigarette:   - ever: 25.8%   - former: 8.8%   - current: 17% - Cigar (ever):   - men 25%   - women8% - Pipe smoking (ever):   - men 16% |
| Behbehani 2004 [37] | - Sampling method: simple random sample in Kuwait and all physicians in Bahrain - Validity of tool: self developed tool, validation not reported (based on a previously reported validated WHO questionnaire for health professionals [67] - Response rate: 46% in Kuwait , 81.6% in Bahrain | Country: Kuwait and Bahrain   - Population: physicians in Kuwait (67% males; mean age 44.6) and Bahrain(61% males; mean age 44.7) - Setting: physician offices, May 2000- Mar 2001. - N sampled:2306 in Kuwait and 644 in Bahrain - N participated: 1059 in Kuwait and 470 in Bahrain - N analyzed: 1059 in Kuwait and 470 in Bahrain | - Waterpipe (daily or occasional):   Kuwait: 12%   - - male: 16.8%   - female: 3.3%   Bahrain: 6.4%   - - male: 8.5%   - female: 2.9% - Cigarette (daily or occasional)   Kuwait: 18.4%   - - male: 24%   - female: 4.3%   Bahrain: 14.6%   - - male: 20.5%   - female: 1.6% |
| Taha 2007 [19] | - Sampling method: multistage stratified random sampling; stratification by government vs. private school, intermediate or secondary level; classes randomly selected - Validity of tool: not reported - Response rate: 100% | - Country: Saudi Arabia - Population: Al Khobar area school male students (mean age 16.5) and their male teachers (mean age 35.1) - Setting: schools classes, end of 2001 - N sampled:1240 students and 142 teachers - N participated: 1240 students and 142 teachers - N analyzed: 1240 students and 142 teachers | - Waterpipe (students)   - current 8.7%,   - former 4% - Waterpipe (teachers)   - current 4.2%,   - former 0.7% - Cigarette (students):   - current 13.1%,   - former 8.1% - Cigarette (teachers):   - current 16.9%,   - former 9.9% |
| AL-Turki 2006 [24] | - Sampling method: simple random sampling - Validity of tool: not reported - Response rate: 80.5% | - Country: Saudi Arabia - Population: male medical students of College of Medicine, King Saud University (mean age 21.8) - Setting: college of medicine, Sep 2005 - N sampled:400 - N participated:322 - N analyzed:322 | - Waterpipe: 8.1% (ever) - Waterpipe and cigarette: 4.4% (ever) - Cigarette: 5.9% (ever) |
| Milaat 1999 [36] | - Sampling method: multistage sampling - Validity of tool: not reported - Response rate: not reported | - Country: Saudi Arabia - Population: KAU female teaching staff and employees in colleges, deanships and administrations (age range 23-62 years) - Setting: not reported - N sampled:299 - N participated:299 - N analyzed:299 | - Waterpipe:   - current: 11% - Cigarette:   - current 10%   - ex smoker 3% |
| Mandil 2007 [25] | - Sampling method: stratified random sampling; stratification by colleges of UoS - Validity of tool: self developed tool, validation not reported (based on previously developed validated WHO questionnaire [68] and GYTS questionnaire [69] - Response rate: 82% | - Country: United Arab Emirates - Population: students in 13 colleges of UoS (39.3% males; mean age 20.9) - Setting: lecture halls, academic year 2004/05 - N sampled:1290 - N participated: 1057 - N analyzed: 1057 | - Waterpipe: 5.8%   - male: 10.8%   - female: 2.5% - Cigarette: 9.4%   - male: 22.2%   - female: 1.4% |
| Al-Mulla 2008 [20] | - Sampling method: 2 stage cluster sampling - Validity of tool: GYTS tool [70] measures “forms of smoked tobacco products other than cigarettes” which the authors assumed to be waterpipe smoking. - Response rate: Bahrain 95.2%, Kuwait 94.8%, Oman 96.9%, Qatar 84.5%, Saudi Arabia 87.5%, United Arab Emirates 95.1%, Yemen 84.3% | - Countries: 7 members states of HMC/GCC - Population: students in grades associated with ages 13-15 (both male and female) except in Saudi Arabia (boys schools in Riyadh) - Setting: schools, 2001-2004 - N sampled: 36302 - N participated: 32356 - N analyzed: 32356 | - Waterpipe (current):   - Bahrain: 15.3%,   - Kuwait: 16.2%   - Oman: 9.4% ,   - Qatar: 13.7%,   - Saudi Arabia (boys only): 10.3%   - UAE: 15%,   - Yemen: 14.6% - Cigarette (current):   - Bahrain: 10.6%,   - Kuwait: 10%,   - Oman: 6.8%,   - Qatar: l 6.4%,   - Saudi Arabia (boys only): 4.7%   - UAE: 6.8%,   - Yemen: 5.3%, - Any form (current):   - Bahrain: 19.9% ,   - Kuwait: 21.1% ,   - Oman:14.3%,   - Qatar: 16.6%,   - Saudi Arabia (boys only): 13.2%   - UAE: 18.5%,   - Yemen: 17.7%, |
| Ward 2006 [38] | - Sampling method: two-stage, stratified, cluster sampling; weighted sampling. Stratification by neighborhood - Validity of tool: not reported - Response rate: 86% | - Country: Syria - Population: adults 18– 65 years in Aleppo (45.2% men; mean age 35.3) - Setting: 2004, location not reported. - N sampled: not reported - N participated: 2038 - N analyzed: 2038 | - Waterpipe:   - current: 11.6%   - daily 1%   - occasionally 10.6% - Cigarette:   - current: 34.5%   - daily 29%   - occasionally 5.5% - Both waterpipe and cigarette: 7.4% - Combined daily use of waterpipe and cigarette: 0.1% |
| Maziak 2005 [39] | - Sampling method: multistage stratified sampling; stratification by type of neighborhood - Validity of tool: self developed tool, validation not reported (based on standardized questionnaires used in international settings as well as those used by Maziak et al in Syria[60, 61] - Response rate: 86% | - Country: Syria - Population: adults aged 18-65 residing in Aleppo “informal zones”, (46% males; mean age 34) - Setting: households, timing not reported - N sampled: not reported - N participated: 1021 - N analyzed: 1021 | - Waterpipe (current): 9.1%   - male: 16%   - female: 4% - Cigarette (current): 39.5%   - male: 62%   - female: 21% |
| Maziak 2004 [9, 26-28] | - Sampling method: multistage sampling - Validity of tool: self developed tool, validation not reported (based on a standardized instrument from the WHO (IUTLD) [69], and from previous questionnaires used in Syria [60] - Response rate: 98.8% | - Country: Syria - Population: Aleppo University students of all fields and grades (47.4% males; mean age 21.8) - Setting: dormitories, 2003 - N sampled: not reported - N participated: 587 - N analyzed: 587 | - Waterpipe:   - current: 14.65%   male 25.5%, female 4.9%   - - ever: 45.6%   male 62.6%, female 29.8%   - - daily: 0.85%   male 1.8%  female 0%   - - occasional: 13.8%   male 23.7%  female 4.9%   - Cigarette:   - current: 18.56%   male: 30.9%  female: 7.4% |
| Gadalla 2003 [29] | - Sampling method: multistage sampling. - Validity of tool: self developed tool, validation not reported - Response rate: 90.7% | - Country: Egypt - Population: students of secondary schools in villages of Qualyobia governorate - Setting: classrooms, academic year 2002/03 - N sampled: 700 - N participated: 635 - N analyzed: 627 | - Waterpipe (life time): 19%   - male: 26%   - female: 5% - Cigarette (lifetime): 29% |
| Habib 2000 [30] | - Sampling method: systematic random sample. - Validity of tool: self developed tool, validation not reported.   Response rate: not reported. | - Country: Egypt - Population: males in households of the village Aghour el Soughra. - Setting: houses, 1997 - N sampled: not reported - N participated: 1827 - N analyzed: 1827 | - Group water pipe smoking: (males only) 10.6% |
| El Sadawy 2004 [31] | - Sampling method: multistage stratified random sampling; stratification by urban vs. rural - Validity of tool: not reported; “a pre designed questionnaire”.   Response rate: 100% | - Country: Egypt - Population: males in household of the village and cities in Sharkia Governorate - Setting: houses, timing not reported. - N sampled: 782 - N participated: 782 - N analyzed: 782 | - Group water pipe smoking (males only): 14.6% |
| Medhat 2002 [32] | - Sampling method: targeted sampling - Validity of tool: self developed tool, validation not reported.   Response rate: not reported. | - Country: Egypt - Population: male village inhabitants 5 years and older - Setting: location and timing not reported. - N sampled: not reported - N participated: 2717. - N analyzed: 2717 | - Group water pipe smoking (males only): 12.5% - Group water pipe smoking: (> 30 years old males): 7.2% |

**Tables 2:** Characteristics of included studies measuring waterpipe smoking prevalence in South Asia

| **Study ID** | **Methodology** | **Population/Setting** | **Prevalence Results** |
| --- | --- | --- | --- |
| Jawaid 2008 [43] | - Sampling method: stratified multistage random sampling; stratification by medical vs. non-medical with systematic sampling of students - Validity of tool: self developed tool, validation not reported - Response rate: 92% | - Country: Pakistan - Population: University students in Karachi (59.6% males; mean age 21) - Setting: Universities, Mar 2006 – Mar 2007 - N sampled: 487 - N participated: 450 - N analyzed: 450 | - Waterpipe (current): 33.1%   - < 1/month: 18.7%   - ≥ 1/month: 10%   - ≥ 1/week: 3.3%   - daily: 1.1% - Waterpipe (ever):   - overall 53.6%   - male: 64.2%   - female: 37.9% - Sharing waterpipe: 31.1% |
| Nisar 2007 [45] | - Sampling method: systematic random sampling - Validity of tool self developed tool, validation not reported (semi structured questionnaire) - Response rate: not reported | - Country: Pakistan - Population: adults above 18 years of age living in a semi urban community of Gadap town, Karachi (63.7% males) - Setting: a house-to-house survey, Jun to Aug 2005 - N sampled: not reported - N participated: 157 - N analyzed: 157 | - Waterpipe: 13.4% - Cigarette: 27.4% - Pan (betel nut): 29.3% - Any form: 70% |
| Nisar 2005 [44] | - Sampling method: systematic random sampling - Validity of tool: self developed tool, validation not reported (semi structured questionnaire) - Response rate: not reported | - Country: Pakistan - Population: adult females above 18 years of age from a community in Manora Island, Karachi - Setting: households, timing not reported. - N sampled: 200 - N participated: 200 - N analyzed: 200 | - Waterpipe: 41% - Cigarette: 2.5% - Niswar (oral dip): 3% - Pan(betel nut): 5.5% - Any form: 52% |
| Alam 1998 [46] | - Sampling method: two stage stratified sampling; stratification by blocks in urban areas and villages in rural areas - Validity of tool: not reported - Response rate (by household): 97.9% | - Country: Pakistan - Population: Adults (15 years and above) living in urban and rural area of Pakistan (46.8%males) - Setting: 1990-94, location not reported - N sampled: not reported - N participated: 9441 - N analyzed: 9441 | - Waterpipe: 6.4%   - male: 7.4%   - female: 5.6% - Cigarette/Beedi: 11.5%   - male: 21.6%   - female: 2.6% - Both waterpipe and cigarette/beedi: 3.6%   - male: 6.9%   - female: 0.8% - Any form: 21.6%   - male: 36%   - female: 9% - Mean age of smoking initiation:   Waterpipe:   - - male: 20   - female: 29   Cigarettes:   - - male: 18   - female: 24 - Mean consumption:   Waterpipe:   - - male: 5 times/day   - female: 4 times/day   Cigarettes:   - - male: 10/day   - female: 5/day |

**Table 3:** Characteristics of included studies measuring waterpipe smoking prevalence in the Americas

| **Study ID** | **Methodology** | **Population/Setting** | **Prevalence Results** |
| --- | --- | --- | --- |
| Primack 2008 [51] | - Sampling method: random sampling - Validity of tool: self developed tool, validation not reported (8 questions added to the National College Health Assessment (NCHA) tool [71] - Response rate: 17.97% | - Country: USA - Population: graduate and undergraduate students of University of Pittsburg (mean age 20.9; 34.4% males) living on and off campus - Setting: online survey, Apr 2007 - N sampled: 3600 - N participated: 660 - N analyzed: 647 | - Waterpipe:   - past 30 days: 9.5%   - ever: 40.5%   - past year: 30.6% - Cigarette:   - past 30 days: 21.5%   - ever: 39.6% |
| Weglicki 2008 [47, 48] | - Sampling method: targeted sampling - Validity of tool: self developed tool, validation not reported (6 questions added to Youth Risk Behavior Surveillance Survey (YRBSS) [72]   Response rate: > 99.9% | - Country: USA - Population: Arab-American and non–Arab-American youth (14–18) able to read and write English. - Setting: high schools; 2004/05 - N sampled: not reported - N participated: 2504 - N analyzed: 1872 | - Waterpipe (current):   - Overall: 15.1%   - Arabs: 16.7%   - Non Arabs: 11.3% - Cigarette (current):   - Overall: 11.4%   - Arabs 6.9%   - Non Arabs: 21.9% |
| Rice 2007 [49] | - Sampling method: targeted sampling - Validity of tool: self developed tool, validation not reported (based on previously reported validated tool: Tobacco Use Questionnaire (TUQ) [73] - Response rate: 100% | - Country: USA - Population: Arab American and non-Arab American youth attending Community High School in Michigan (mean age 14.5; 55% males) - Setting: location and timing not reported - N sampled: 1455 - N participated: 1455 - N analyzed: 1455 | - Waterpipe (Arab youth):   - last 30 days 12%   - experimental 36%   - regular 8% - Waterpipe (non-Arab youth):   - last 30 days 4%   - experimental 11%   - regular 3% - Cigarette (Arab youth):   - last 30 days 2%   - experimental 9%   - regular 1% - Cigarette (non –Arab youth):   - last 30 days 9%   - experimental 27%   - regular 5% |
| Rice 2006 [11] | - Sampling method: targeted sampling - Validity of tool: self developed tool, validation not reported (based on previously reported validated tool: Tobacco Use Questionnaire (TUQ) [73] - Response rate: 96.22% from school, 45% from the clinic | - Country: USA - Population: adolescents 14 to 18 years old (mean age 15.4, 52% males), able to read and write in English or Arabic with one or more parents of Arab origin - Setting: teen health clinic and a school, school year 2001/02. - N sampled: 2225 - N participated: 1671   N analyzed: 1671 | - Waterpipe:   - current: not reported   - experimentation: 27%   - ever by age 14: 23%   - ever by age 18: 40% - Cigarette:   - current: 6.9%   - experimentation: 29%   - ever by age 14: 15%   - ever by age 18: 44% - Many youth had first used the waterpipe before the age of 10 |
| Ward 2006 [52] | - Sampling method: all recruits participated in the study - Validity of tool: not reported - Response rate: 100%, follow up rate 86.2% | - Country: USA - Population: active duty Air Force personnel entering the US Air Force for Basic Military Training (BMT) - Setting: baseline data (Oct 1999 -Oct 2000) and at 12 month follow-up data (late 2000 - early 2002) - N sampled: 20,673 - N participated: 20,673(baseline) - N analyzed: 20,673 (baseline) | - Waterpipe use: 0.3% - Cigarettes use: 29.9% |
| Primack 2009 [50] | - Sampling method: stratified 2-stage cluster sampling, weighted sampling - Validity of tool self developed tool, validation not reported (2 questions added to the Arizona Tobacco Survey YTS questionnaire [74] - Response rate: 80% | - Country: USA - Population: 6 through 12 grade students of Arizona district and charter schools (48.1% males; median age 14) - Setting: classrooms, spring semester of academic year 2004/05 - N sampled: 7,646 - N participated: 6,594 - N analyzed: 6,594 | - Waterpipe (past 30 days): 3.5%   - middle school students: 1.4%   - high school students: 5.4%   - 12th graders: 7.3% - Waterpipe (ever): 6.4%   - middle school students: 2.1%   - high school students: 10.3%   - 12th graders: 15.1% |

**Tables 4:** Characteristics of included studies measuring waterpipe smoking prevalence in Europe

| **Study ID** | **Methodology** | **Population/Setting** | **Prevalence Results** |
| --- | --- | --- | --- |
| Jackson 2008 [12] | - Sampling method: random cluster sampling - Validity of tool: not reported - Response rate: not reported | - Country: United Kingdom - Population: students of University of Birmingham (41.9% males) - Setting: lecture halls, timing not reported - N sampled: not reported - N participated: 937 - N analyzed: 937 | - Waterpipe (ever): 37.9%   - regular: 8%   - ≥ weekly: 4.2%   - daily: 1.7% - Waterpipe only: 4.8% - Waterpipe with other form of tobacco : 3.2% - Any form: 15.9% - Cigarette only: 5.4% - Cigar only: 0.3% - Chew tobacco only: 0.96% - More than one form of tobacco: 4.4% |
| Parna 2008 [53] | - Sampling method: multistage random sampling; stratification by geographic region, urbanization, and study language in the school, cluster was class - Validity of tool: self developed tool, validation not reported (1 question addressing waterpipe added to a previously validated instrument, Health Behavior in School-aged Children survey (HBSC) of WHO [61] - Response rate: not reported | - Country: Estonia - Population: 11-15 year old school students (49.5% males) - Setting: classrooms, Oct 2006-May 2007 - N sampled: not reported - N participated: 4463 - N analyzed: 4463 | - Waterpipe (current): 20.7%   - male: 25.2%   - female: 16.2% - Waterpipe (daily): 0.8%   - male: 1.3%   - female: 0.3% - Waterpipe (≤1/week): 16.5%   - male: 19.3%   - female: 13.8% - Waterpipe (>1/week): 3.4%   - male: 4.6%   - female: 2.1% - Cigarette (current): 15.6%   - male: 18.3%   - female: 13% |

**Tables 5:** Characteristics of included studies measuring waterpipe smoking prevalence in Australia

| **Study ID** | **Methodology** | **Population/Setting** | **Prevalence Results** |
| --- | --- | --- | --- |
| Carroll 2008 [13, 54] | - Sampling method: random sampling - Validity of tool: not reported - Response rate: 70% | - Country: Australia - Population: Arabic speakers in Australia - Setting: Aug-Nov 2004, location not reported - N sampled: 1102 - N participated: not reported - N analyzed: not reported | - Waterpipe (current): 11.4 % - Waterpipe (daily): 1% |
