# Supplementary material for: The prevalence of waterpipe tobacco smoking among the general and specific populations: a systematic review
Source: BMC Public Health. 2011 Apr 19;11:244. doi: 10.1186/1471-2458-11-244 (PMC3100253; doi:10.1186/1471-2458-11-244)
Supplement: Additional file 4 — Results of the Global Youth Tobacco Survey (GYTS) and World Health Organization (WHO) surveys. Provides the prevalence of waterpipe smoking in the GYTS and WHO surveys [file 1471-2458-11-244-S4.DOCX]

**Additional file 4**: Results of the Global Youth Tobacco Survey (GYTS) and World Health Organization (WHO) surveys

| **Country** | **Population** | **Results** |
| --- | --- | --- |
| Iran 2007 * | 13-15 year age group | - Waterpipe (current): 16.5% - male: 22.8% - female: 9.4% - Waterpipe (ever): 43.8% - male: 55% - female: 32% |
| Lebanon 2001 * | 13-15 year age group | - Waterpipe (ever): 62% - male: 67% - female: 58% |
| Egypt 2002 † | Adult males | - Waterpipe only: 10% |
| Egypt 2005 † | Adult males aged 18 and older | - Waterpipe (current): - rural: 13.6% - urban: 10.5% |

* GYTS studies

† WHO
